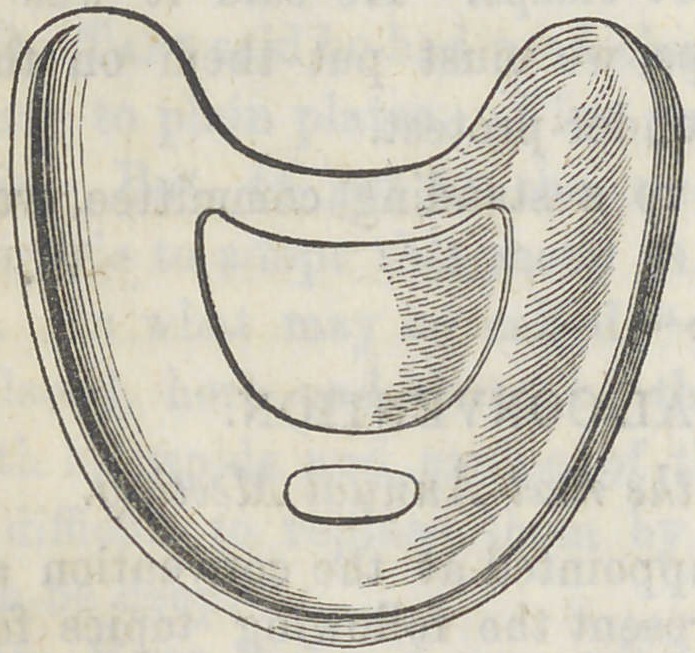# Improved Plates

**Published:** 1858-03

**Authors:** J. W. Winter


### IMPROVED PLATES.

BY DR. J. W. WINTER.

With this I send a rough pencil drawing of an improvement in section plates, for full upper plates. Its advantages are: first, the plate is so formed as to prevent all springing in soldering. Secondly, the patient can draw up the plate more firmly, and in less time, than the ordinary plate.

A hole is cut through the plate just back of the incisors, between them and the chamber. This is the best possible point from which to draw out the air and saliva.

The accompaning illustration gives the position of the chamber and hole.

I trust others will investigate this matter thoroughly. I have been using this kind of plates for the last four years with success. I consider it a great improvement in mechanical dentistry. I am indebted for the improvement to Dr. Waters, in 1851.

The accompaning cut illustrates Dr. Winter's idea fully There are some cases in which this method can be employed with decide advantage, others again, in which it would be of no advantage, but probably injurious. In cases when the soft parts are thick, and soft, there would be danger of the mucous membrane protruding through the opening, and becoming irritated. In cases where the arch is very deep and rough; in which it would be difficult to adapt the plate to every point, I think the principle would not be applicable. We hope every one will experiment with it and report. Ed.

## Figures and Tables

**Figure f1:**